# Enhancement of Tobacco (*Nicotiana tabacum* L.) Seed Lipid Content for Biodiesel Production by CRISPR-Cas9-Mediated Knockout of *NtAn1*

**DOI:** 10.3389/fpls.2020.599474

**Published:** 2021-01-21

**Authors:** Yinshuai Tian, Xinanbei Liu, Caixin Fan, Tingting Li, Huan Qin, Xiao Li, Kai Chen, Yunpu Zheng, Fang Chen, Ying Xu

**Affiliations:** ^1^Key Laboratory of Bio-Resources and Eco-Environment of Ministry of Education, College of Life Sciences, Sichuan University, Chengdu, China; ^2^School of Landscape and Ecological Engineering, Hebei University of Engineering, Handan, China; ^3^Institute of New Energy and Low-Carbon Technology, Sichuan University, Chengdu, China; ^4^School of Water Conservancy and Hydroelectric Power, Hebei University of Engineering, Handan, China

**Keywords:** tobacco, seed lipid, NtAn1, CRISPR-Cas9, biodiesel

## Abstract

Tobacco (*Nicotiana tabacum* L.) seed lipid is a promising non-edible feedstock for biodiesel production. In order to meet the increasing demand, achieving high seed lipid content is one of the major goals in tobacco seed production. The *TT8* gene and its homologs negatively regulate seed lipid accumulation in *Arabidopsis* and *Brassica* species. We speculated that manipulating the homolog genes of *TT8* in tobacco could enhance the accumulation of seed lipid. In this present study, we found that the *TT8* homolog genes in tobacco, *NtAn1a* and *NtAn1b*, were highly expressed in developing seed. Targeted mutagenesis of *NtAn1* genes was created by the CRISPR-Cas9-based gene editing technology. Due to the defect of proanthocyanidin (PA) biosynthesis, mutant seeds showed the phenotype of a yellow seed coat. Seed lipid accumulation was enhanced by about 18 and 15% in two targeted mutant lines. Protein content was also significantly increased in mutant seeds. In addition, the seed yield-related traits were not affected by the targeted mutagenesis of *NtAn1* genes. Thus, the overall lipid productivity of the *NtAn1* knockout mutants was dramatically enhanced. The results in this present paper indicated that tobacco *NtAn1* genes regulate both PAs and lipid accumulation in the process of seed development and that targeted mutagenesis of *NtAn1* genes could generate a yellow-seeded tobacco variety with high lipid and protein content. Furthermore, the present results revealed that the CRISPR-Cas9 system could be employed in tobacco seed *de novo* domestication for biodiesel feedstock production.

## Introduction

Due to the increasing concerns about climate change resulting from excessive consumption of petroleum products, biodiesel has attracted more and more attentions in the recent years. Tobacco (*Nicotiana tabacum* L.) is an oilseed plant with a high seed lipid content ranging from 36 to 41% of the seed dry weight (Zlatanov et al., 2007; Usta et al., 2011). Recently, tobacco seed lipid had been demonstrated to be a promising feedstock for biodiesel production (Giannelos et al., 2002; Veljkovic et al., 2006; Banković-Ilić et al., 2012; Kumar and Sharma, 2016). Furthermore, due to the rich carbohydrates, wide availability, and low cost, tobacco stalk could also be used for biofuel production (Cai et al., 2016). Recently, a high seed yield tobacco variety, Solaris, had been bred by the Sunchem Holding Company for seed lipid feedstock production (Grisan et al., 2016). Life cycle analysis showed that the impacts created by the production of Solaris tobacco biodiesel were similar to those from other biodiesel plants (Carvalho et al., 2019). Sustainable provision of feedstock is the key to sustainable biofuels (Poltronieri, 2016). In order to meet the huge demand for the feedstock of biodiesel production, achieving high tobacco seed lipid content is one of the main goals in the future.

Sucrose produced by photosynthetic tissues serves as a major carbon source for the synthesis of both seed storage compounds and the generation of other seed components such as mucilage and proanthocyanidins (PAs, also called as condensed tannins) in seed coat. Seed coat development competes for sucrose with reserve component synthesis in embryo and endosperm. Recently, studies had demonstrated that the amount of PAs in the seed coat is negatively correlated with the amount of lipid content in the embryo in *Arabidopsis* and rapeseed (Wang et al., 2014; Zhai et al., 2019). Thus, strategies of manipulating the PA biosynthesis pathway in seed coat could be used to increase the seed lipid content.

Proanthocyanidin deposit in the inner integument of the seed coat, and the oxidation of PAs in the process of seed maturation results in the formation of brown pigments that confer color to the mature seed (Lepiniec et al., 2006). Previous studies had demonstrated that the biosynthesis of PAs were mainly regulated at the transcription level by transcription factors belonging to the basic helix-loop-helix (bHLH), R2R3-MYB, and WD-repeat protein families (Lepiniec et al., 2006; Xu et al., 2015b). In *Arabidopsis*, *TT2*, *TT8*, and *TTG1*, which encode R2R3-MYB, bHLH, and WD40 repeat proteins, respectively, form a ternary complex to activate the expression of PA-specific genes during seed coat development (Baudry et al., 2004; Xu et al., 2015b).

Previous studies revealed that *TT8* gene played a key regulation role in seed PA biosynthesis. In *Arabidopsis*, the *TT8* gene is required for the expression of *DFR* and *BAN* genes in siliques and young seedlings (Nesi et al., 2000). Due to the defect of PAs synthesis, *Arabidopsis tt8* mutants showed the transparent test of a phenotype (Nesi et al., 2000). In addition to *Arabidopsis*, natural mutation of *TT8* genes resulted in yellow seed coat trait in allotetraploid *Brassica juncea* (Padmaja et al., 2014). Most recently, targeted mutation of the *TT8* homologs through the CRISPR-Cas9 system in *Brassica napus* also generated a yellow-seeded phenotype (Zhai et al., 2019). Homologs of *TT8* gene were also reported to be involved in PA biosynthesis in diverse plant species, including *Medicago truncatula*, *Lotus corniculatus*, and *Raphanus sativus* (Li et al., 2016; Escaray et al., 2017; Lim et al., 2017).

In addition to its critical role in PA biosynthesis regulation, the *Arabidopsis* TT8 protein could repress the seed lipid accumulation by inhibiting the expression of transcription factors, including *LEC1*, *LEC2*, and *FUS3*, which play the key roles in embryo development and seed lipid biosynthesis (Chen et al., 2014). Furthermore, TT8 protein could directly repress the expression of genes encoding enzymes involved in fatty acid biosynthesis by binding to the promoter region (Chen et al., 2014). Thus, the mutation of *TT8* gene generated seed with a thinner seed coat, a reduced PAs content, and an increased content of lipid in *Arabidopsis* and *Brassica* species (Li et al., 2012; Chen et al., 2014; Zhai et al., 2019). We suggested that *TT8* gene could be a promising target aimed at enhancing the lipid content for an oilseed plant.

Due to the presence of PAs, tobacco seed shows a dark brown seed color. Thus, we proposed that *TT8* homolog genes in tobacco might be an ideal candidate to create a high lipid content seed for biodiesel production. Previous report had identified and characterized two *TT8* homolog genes in tobacco genome, *NtAn1a* and *NtAn1b*, originated from two ancestors of tobacco, *Nicotiana sylvestris* and *Nicotiana tomentosiformis*, respectively (Bai et al., 2011). *NtAn1* genes were demonstrated to be involved in flower anthocyanin biosynthesis (Bai et al., 2011); however, if *NtAn1* genes regulate the accumulation of PAs and lipid during seed development have not been reported. In this present paper, the expression pattern of *NtAn1* genes during seed development was analyzed, and the CRISPR-Cas9 system was applied to generate *NtAn1* knockout mutants. We found that the targeted mutagenesis of *NtAn1* genes significantly enhanced the accumulation of seed lipid and protein. These results demonstrated that the CRISPR-Cas9-mediated knockout of *NtAn1* genes is an efficient approach to improve lipid production in tobacco.

## Materials and Methods

### Plant Material and Growth Condition

Wild-type (WT) tobacco cultivar (*Nicotiana tabacum* L. “K326”) was used for gene expression analysis and genetic transformation. Tobacco seeds were surface-sterilized with 75% ethanol for 5 min and washed three times with absolute ethanol. Then, the surface-sterilized seeds were germinated on 1/2 MS medium containing 2% sucrose and 0.7% agar at 25 ± 1°C. The seedlings were transferred to soil and grown in greenhouse at 25 ± 1°C with a photoperiod of 16-h light/8-h dark.

### Quantitative Real-Time PCR

Total RNA was isolated using Plant Total RNA Isolation Kit (Foregene, China). PrimeScript™ RT reagent Kit (Takara, Dalian) was used to generate cDNA according to the manufacturer’s instruction. Quantitative real-time PCR (qRT-PCR) was carried out using SYBR Premix Ex Taq™ II (Takara, Dalian) on a CFX96 real-time PCR system (Bio-Rad, United States). The qRT-PCR cycling began with one cycle at 95°C for 2 min, followed by 40 cycles at 95°C for 30 s, 55°C for 30 s, and 72°C for 30 s. The house-keeping gene, *NtGAPDH* (XM_016643257), was used as the reference standard. Three biological and three technical replicates were performed. Primers used for qRT-PCR analysis are listed in Supplementary Table S1.

### Vector Construction

To generate knockout mutants using the CRISPR-Cas9 system, the pKSE401 vector (purchased from Addgene: #62202) was used for tobacco genetic transformation. Two guide RNAs (gRNA) targeting the second extron of *NtAn1* coding region were designed using CRISPR-P 2.0 (Liu et al., 2017).^1^ The scaffold containing two gRNAs was amplified by PCR using a pCBC-DT1T2 vector (purchased from Addgene: #50590) as a template. The PCR product was purified using the Universal DNA Purification Kit (TIANGEN, China) and inserted into the pKSE401 vector using a Golden-Gate Assembly method as described previously (Xing et al., 2014). The reconstructed vector was introduced into the DH5*α* strain of *Escherichia coli* and confirmed using a Sanger sequencing (Sangon Biotech, China). The verified vector (Named as *pKSE401-An1*) was introduced into *Agrobacterium tumefaciens* strain GV3101 for tobacco genetic transformation. Primers used for vector construction are listed in Supplementary Table S2.

### Tobacco Transformation and Mutant Selection

Agrobacterium-mediated tobacco leaf disc transformation experiment was performed as previously described (Horsch, 1985). T0 generation transgenic lines were selected on mass spectrometry (MS) medium supplemented with 50 mg/l kanamycin. Genomic DNA was extracted from the kanamycin-resistant T0 transgenic lines using a Super Plant Genomic DNA Kit (TIANGEN, China). To select mutant lines, the flanking region of the gRNAs targeting sites was amplified using sequence-specific primers by PCR (primers are listed in Supplementary Table S2). The PCR products were purified and sequenced immediately by the Sanger method (Sangon Biotech, China). The T0 mutant lines were self-pollinated to generate T1 seeds. The T1 plants were analyzed again to confirm the mutation. In addition, the presence of the CRISPR-Cas9 construct in T1 mutant plants was examined by PCR using vector specific primers (primer sequences are listed in Supplementary Table S2). The homozygous T1 mutant plants without the CRISPR-Cas9 construct were used for further analysis.

### DMACA Staining

To visualize the accumulation of PAs in seed coat, dry mature tobacco seeds from WT and mutant plants were stained in a freshly prepared dimethylaminocinnamaldehyde (DMACA, Sigma, United States) reagent [2% (w/v) DMACA dissolved in 6 N HCl/95% ethanol mixture (1:1, v/v)] for 30 min and then washed several times with 70% ethanol (v/v) as described previously (Hong et al., 2017). The stained seeds were photographed using a Leica stereomicroscope (Leica, Germany).

### PAs Quantification

Quantification of PAs was performed according to the previous report (Pang et al., 2008). Briefly, to extract soluble PAs from tobacco seeds, 200 mg of dry seeds were ground in liquid nitrogen and extracted with 1 ml extraction solution (70% acetone/0.5% acetic acid) by vortexing for 10 s. After sonication at room temperature for 1 h, the mixture was centrifuged at 2,500 *g* for 10 min, and the residue was re-extracted twice. The pooled supernatants were extracted twice with hexane. To quantify the soluble PAs level, 50 μl of the supernatant sample was mixed with 200 μl of DMACA reagent (0.1% DMACA, 90% ethanol, 10% HCl) in 96-well plates, and the absorption was measured at 640 nm. Soluble PA levels were calculated using a standard curve prepared using procyanidin B1 (Sigma, United States).

The residue from soluble PAs extraction was air dried and used for quantitative analysis of insoluble PAs. Five-hundred microliter butanol-HCl reagent (95% butanol:5% concentrated HCl) was added to the residue, and the mixture was sonicated at room temperature for 1 h, followed by centrifugation at 2,500 *g* for 10 min. The absorption of the supernatant was measured at 550 nm, then samples were boiled for 1 h and cooled to room temperature, and the absorbance at 550 nm was recorded again, with the first value being subtracted from the second. Absorbance values were converted into PA equivalents using a standard curve of procyanidin B1 (Sigma, United States).

### Seed Lipid Assay

To determine the seed lipid content and fatty acid composition, fatty acid methyl esters (FAMEs) were prepared as previously described (Li et al., 2006). Twenty mature tobacco seeds were added into the methyl esterification solution [1 ml of 5% sulfuric acid in methanol (v/v), 25 μl 0.2% butylated hydroxyl toluene solution, and 300 μl toluene. Twenty microgram triheptadecanoin was added as internal standard]. The mixture was heated at 90°C for 2 h. Then, 1.5 ml of 0.9% NaCl and 1 ml hexane were added after the mixture had cooled down to room temperature. The FAMEs were separated by collecting the organic phase. The FAMEs were quantitatively analyzed by gas chromatography mass spectrometry (GC–MS; DAOJING, Japan). The GC conditions were as follows: 1 μl injection volume, split injection (1:20), injector temperature 220°C, oven temperature program: 150°C for 1 min, then increased to 200°C at 10°C min^−1^, holding at 200°C for 1 min, and then increased to 210°C at 5°C min^−1^ and held for 1 min. The seed lipid content was quantified according to the peak area of the internal standard.

### Protein Assay

To evaluate the seed protein content, the total protein was extracted as previously described with some modification (Heath et al., 1986). Briefly, 10 mature tobacco seeds were grounded in protein extraction solution [63 mM Tris buffer, pH 7.8, 0.5 M NaCl, and 0.07% (v/v) *β*-mercaptoethanol]. The homogenate samples were centrifuged at 12,000 rpm for 10 min. Twenty microliter of the supernatant was used for protein quantification using the Pierce BCA Protein Assay Kit (Thermo, United States) according to the manufacturer’s protocol. The experiment was repeated three times.

### Yeast One-Hybrid Assay

Yeast one-hybrid assay was performed to investigate if An1b protein could bind to the promoter of the anthocyanidin reductase (ANR) gene. The promoter sequence of ANR gene (574 bp upstream of the start codon) was amplified by PCR using primers pANR-F and pANR-R (primer sequences were listed in Supplementary Table S2) and inserted into the yeast expression vector pHIS2 using the ClonExpress II One Step Cloning Kit (Vazyme, China) to generate pHIS2-ANR. The An1b protein coding region was amplified by PCR using An1b-F and An1b-R (primer sequences are listed in Supplementary Table S2) and cloned into the pGADT7 vector using the ClonExpress II One Step Cloning Kit (Vazyme, China) to generate pGADT7-An1b. The coding region of An2 protein was also amplified using An2-F and An2-R (primer sequences are listed in Supplementary Table S2) and firstly inserted into pDR195 vector using the ClonExpress II One Step Cloning Kit (Vazyme, China). Then, the An2 expression box in pDR195 vector was amplified using An2-box-F and An2-box-R (primer sequences are listed in Supplementary Table S2), and the fragment was inserted into pHIS2-ANR using the ClonExpress II One Step Cloning Kit (Vazyme, China) to obtain pHIS2-ANR + An2. The yeast strain Y187 was co-transformed with pGADT7- and pHIS2- based vectors by the polyethylene glycol-mediated method. The DNA-protein interactions were evaluated according to the growth status of yeast cells cultured on the SD/−Leu/−Trp/-His selective medium with 120mM 3-AT for 3 days at 30°C.

### Yield-Related Traits Assay

For seed size measurement, mature seeds were photographed using a Leica stereomicroscope (Leica, Germany). The seed length and width were measured with the Image J software. For average seed weight analysis, 50 seeds were randomly collected and carefully weighed using an electronic balance (METTLER TOLEDO, United States). Average seed weight was calculated by dividing the total seed weight by the seed number. Fruit number per plant was checked from 20 individual plants at the mature stage. Fruits used for the seed number analysis were obtained from the first five basal fruits of the main inflorescence. The total seed weight from a single fruit was weighed using an electronic balance, and the seed number per fruit was calculated by dividing the total seed weight by the average seed weight.

## Results

### *NtAn1* Genes Were Highly Expressed in Developing Seed

Previous study reported that *Arabidopsis TT8* gene has two homologs in tobacco genome, *NtAn1a* and *NtAn1b* (Bai et al., 2011). Tobacco is a natural allotetraploid plant; the sequence analysis revealed that *NtAn1a* originated from *N. sylvestris*, whereas *NtAn1b* derived from *N. tomentosiformis*. The qPCR analysis showed that *NtAn1a* and *NtAn1b* expressed at developing flowers with the highest expression level in corolla limb, which was consistent with the function of the flower flavonoid biosynthesis regulation (Bai et al., 2011). However, from the previously reported results, we noticed that the transcript level of both *NtAn1a* and *NtAn1b* was relatively high in developing ovary, which indicated that *NtAn1* genes might play an important role in seed development (Bai et al., 2011).

**Figure 1:**
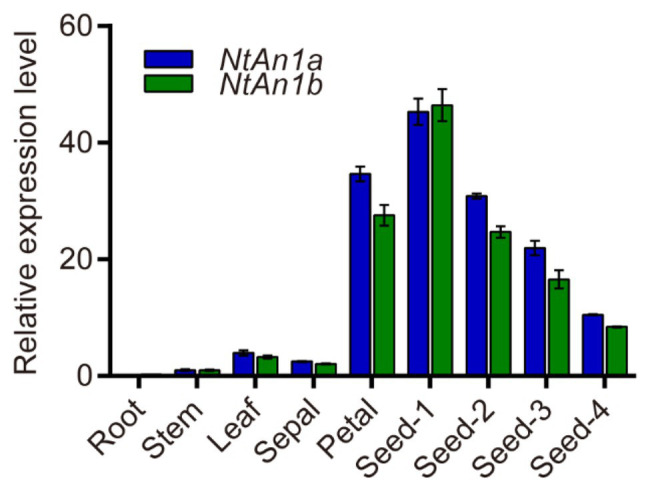
Expression profiles of tobacco *NtAn1a* and *NtAn1b* genes. Seed-1, developing seed at 7 days after flowering (DAF); Seed-2, developing seed at 15 DAF; Seed-3, developing seed at 21 DAF; Seed-4, developing seed at 28 DAF. Data are mean ± SD (*n* = 3). *NtGAPDH* gene was amplified as an internal control.

To further confirm and characterize the expression pattern of *NtAn1a* and *NtAn1b*, their expression was assessed in different organs and various stages of seed development: 7, 14, 21, and 28 days after flowering (DAF). qRT-PCR results showed that *NtAn1a* and *NtAn1b* had a similar expression pattern, with the highest expression in developing seeds at 7 DAF and decreased at later stages (Figure 1). The expression of both *NtAn1* genes exhibited a high expression level in flower, which was consistent with the previous results. However, low transcript levels were detected in root, leaf, and stem (Figure 1). These results suggested that *NtAn1a* and *NtAn1b* might regulate PAs and lipid accumulation during seed development in a way like in *Arabidopsis* and rapeseed.

### Targeted Mutagenesis of *NtAn1* Using the CRISPR-Cas9 System

**Figure 2:**
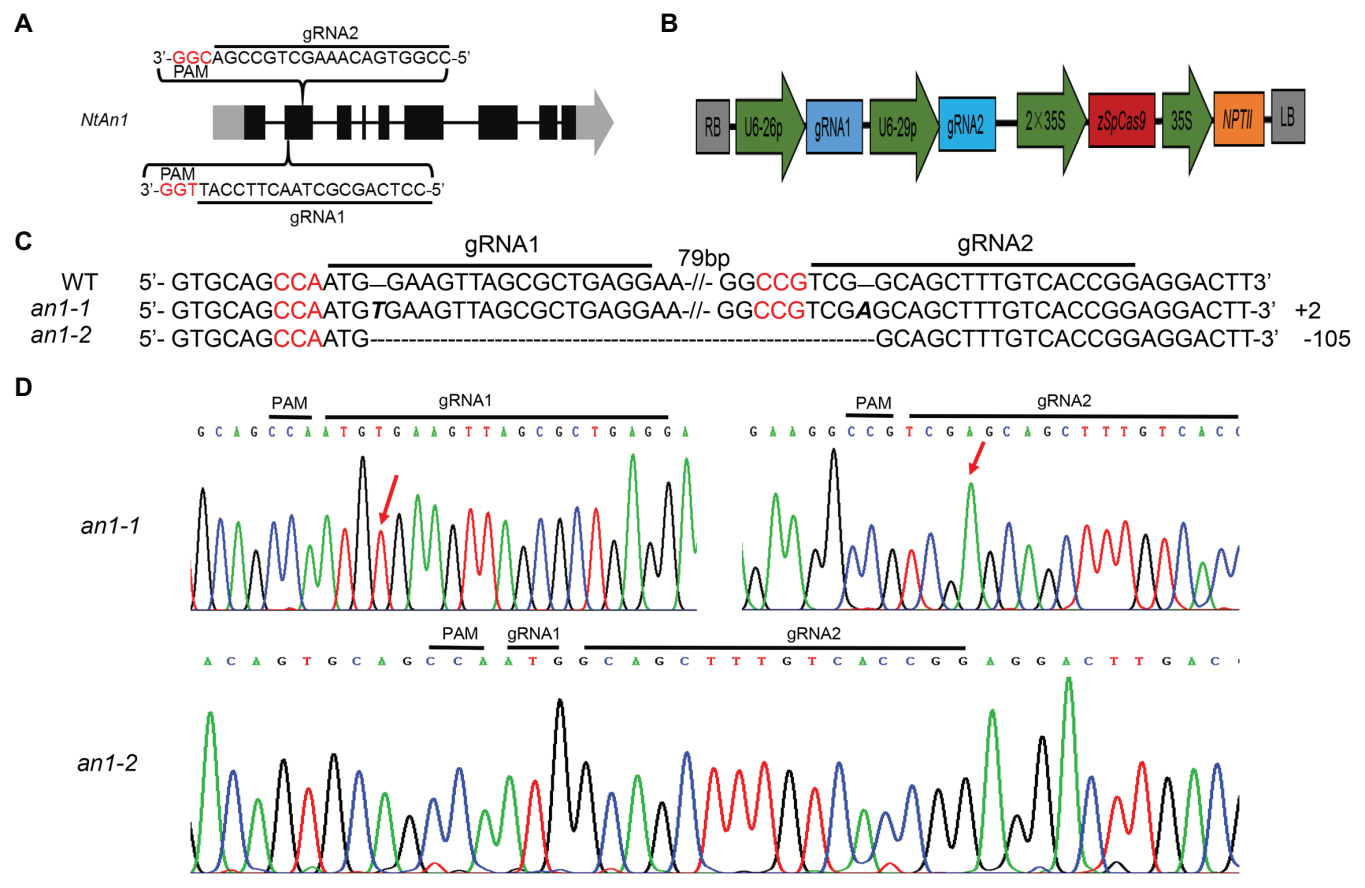
CRISPR-Cas9-mediated targeted mutation of *NtAn1*. **(A)** Targeting sites and gRNA sequences used for *NtAn1* genes editing. The three base protospacer adjacent motif (PAM) was in red. **(B)** The CRISPR-Cas9 vector structure used for targeted mutation of *NtAn1*. **(C)** Mutation type of the *an1-1* and *an1-2* lines determined by the Sanger method. **(D)** Sanger sequencing peaks of the *an1-1* and *an1-2* mutant lines. The red arrows indicate the location of mutations.

*NtAn1a* and *NtAn1b* showed a high sequence identity of 92.95 and 90.36% at the nucleotide and protein levels, respectively (Supplementary Figures S1, S2). Furthermore, these two genes had similar expression patterns (Figure 1). These results suggested that these two genes may have similar and redundant functions. Thus, two gRNAs recognizing both *NtAn1* genes were designed to effectively knockout them and both of the gRNAs targeting the second extron of the coding sequence (Figure 2A). The CRISPR-Cas9 construct containing these two gRNAs, which were driven by the *Arabidopsis* U6-26 and U6-29 promoter, respectively (Figure 2B), was produced based on the CRISPR-Cas9 multiplex genome-editing vector (Xing et al., 2014). The resulting construct was transformed into WT tobacco plant using the Agrobacterium-mediated leaf disc transformation method. Through kanamycin selection, 12 kanamycin resistance T0 transgenic plants were generated. The targeting region of both *NtAn1a* and *NtAn1b* were amplified by a pair of primers at the same time. Two homozygous mutant lines (*an1-1* and *an1-2*) were identified from the T0 transgenic plants by Sanger sequencing analysis of the gRNAs targeting region. The *an1-1* mutant had one base insertion at both gRNA targeting sites of the *NtAn1a* and *NtAn1b* genes, while the *an1-2* mutant line had a 105 base fragment deletion between the two gRNAs targeting sites (Figures 2C,D). T-DNA free mutant plants were selected from the T1 progeny generated by the self-pollinated of two independent T0 homozygous mutant lines. Twenty T-DNA free T1 generation plants from each mutant line were randomly selected for further analysis.

### Mutation of *NtAn1* Genes Resulted in Yellow Seed Coat

**Figure 3:**
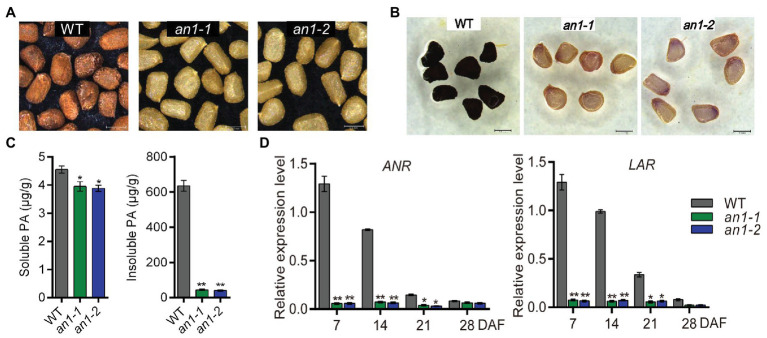
Seed phenotypes of tobacco *an1* mutants. **(A)** Tobacco seed coat color phenotypes of WT and *an1* mutant lines. **(B)** DMACA staining of mature tobacco seeds from WT and *an1* mutant lines. **(C)** Soluble and insoluble PA contents in mature seeds of WT control and *an1* mutant lines. Three biological replicates were analyzed. Data are mean ± SD (*n* = 3). **(D)** Expression of PA biosynthetic genes regulated by *NtAn1* in tobacco. ANR: anthocyanidin reductase and LAR: leucoanthocyanidin reductase. RNA samples were extracted from the developing seeds at 7, 14, 21, and 28 DAF. Data are mean ± SD of three biological replicates. Asterisks indicate significant difference compared to control samples (*p < 0.05; **p < 0.01). *NtGAPDH* gene was used as an internal reference.

The formation of seed color in most plant species is due to the deposition of PAs within the endothelial layer of the inner integument of the seed coat (Lepiniec et al., 2006). Previous studies demonstrated that *TT8* played a key role in regulating PAs accumulation in various plants (Li et al., 2016; Escaray et al., 2017). In this paper, mutation of the *NtAn1*, the *TT8* homolog genes in tobacco, generated a yellow-seeded phenotype (Figure 3A). This indicated that the targeted mutation of the *NtAn1* genes might disrupt the accumulation of PAs in tobacco seed coat. In order to check the PAs deposition visibly, the mutant tobacco seeds were dyed by DMACA reagent. The results further confirmed the defects of PAs accumulation in seed coat (Figure 3B). The soluble and insoluble PAs contents were calculated quantitatively by spectrophotometric method. The results showed that the PAs were mainly stored in the insoluble form in tobacco seed coat, and the targeted mutation of *NtAn1* genes led to the significant decreases in both soluble and insoluble PAs content compared with those in WT tobacco seed coat (Figure 3C).

ANR and leucoanthocyanidin reductase (LAR) are two key enzymes participated in PA biosynthesis. ANR converts anthocyanidins to the epicatechin (Xie et al., 2003), whereas LAR could reduce leucocyanidin to catechin (Tanner et al., 2003). Catechin and epicatechin are considered to be the main building blocks for PAs biosynthesis. The encoding genes of ANR and LAR were directly regulated by *TT8* gene in *Arabidopsis* (Baudry et al., 2004). The expression patterns of the homolog genes encoding these two enzymes during seed development were analyzed by qRT-PCR. Our results showed that both of these two genes had a similar expression profile as *NtAn1*, and the expression of them were significantly decreased in both *an1-1* and *an1-2* mutant seed (Figure 3D). Taken together, these findings indicate that tobacco *NtAn1* regulated the accumulation of PAs in a similar way like in *Arabidopsis*, and the mutation of the tobacco *NtAn1* genes could hinder the PA deposition in the seed coat, which was consistent with the phenotypes observed in *tt8* mutant seed in *Arabidopsis* and other *Brassica* species (Li et al., 2012; Chen et al., 2014; Padmaja et al., 2014; Zhai et al., 2019).

**Figure 4:**
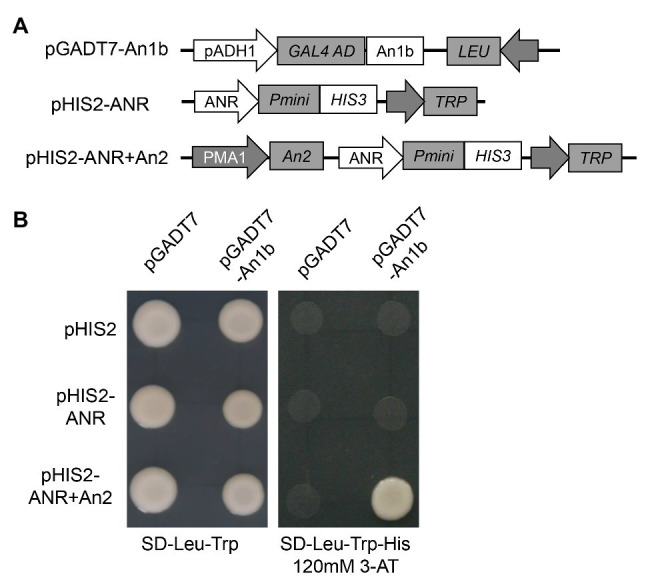
NtAn1 Yeast one-hybrid assay. **(A)** Schematic structure of the vectors used for yeast one-hybrid assay. **(B)** Growth performance on transformed yeast cells on SD/−Leu−/Trp and SD/−Leu−/Trp/-His medium containing 120mM 3-AT.

In order to confirm if the NtAn1 protein could directly bind to the promoter region of the genes involved in PA biosynthesis, yeast one-hybrid assay was performed. The promoter region of ANR gene was inserted into the pHIS2 to generate ppHIS2-LAR as a reporter vector (Figure 4A). Due to the high protein sequence identity of NtAn1a and NtAn1b, the NtAn1b protein was chosen to test the DNA binding ability (Figure 4A). Our result showed that the NtAn1b protein could not bind to the ANR promoter region in yeast cells and activate the HIS reporter gene alone (Figure 4B). Previous study demonstrated that NtAn1 proteins could interact with tobacco An2, a R2R3-MYB family protein (Bai et al., 2011). In addition, the bHLH protein usually forms a MYB-bHLH-WD40 (MBW) ternary complex with WD40 and R2R3-MYB proteins to regulate the expression of downstream PA biosynthesis genes (Baudry et al., 2004; Schaart et al., 2013; Xu et al., 2015b). We speculated that the regulation activity of NtAn1 protein might be dependent on An2. To verify this speculation, the An2 protein was expressed using the pHIS2 vector by introducing an expression box from pDR195 to generate pHIS2-ANR + An2 (Figure 4A). When pGADT7-An1b and pHIS2-ANR + An2 were co-transformed into yeast cells, the HIS reporter gene could be activated (Figure 4B). These results indicated that NtAn1b protein functions together with An2 protein in PA pathway gene regulation. However, the WD40 protein has not been identified so for.

### Targeted Mutagenesis of *NtAn1* Generated White Flower

**Figure 5:**
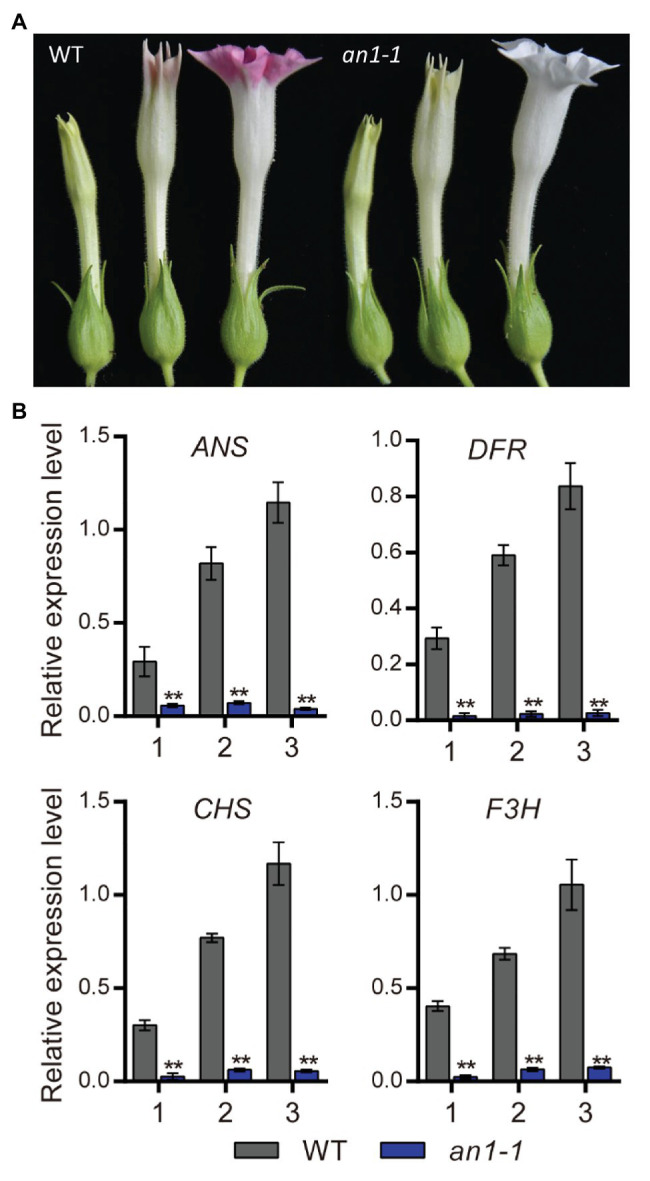
Flower phenotype of tobacco *an1-1* mutant. **(A)** Flower at different development stages from WT and *an1-1* mutant line. **(B)** Expression analysis of anthocyanin biosynthetic genes regulated by *NtAn1* in tobacco. CHS, chalcone synthase; F3H, flavanone 3-hydroxylase; DFR, dihydroflavonol 4-reductase; ANS, anthocyanidin synthase. Data are mean ± SD (*n* = 3). Asterisks indicate significant difference compared to control samples (*p < 0.05; **p < 0.01). *NtGAPDH* gene was used as an internal reference.

Previous study had demonstrated that the anthocyanin accumulation in transgenic tobacco flowers could be significantly elevated by the overexpression of *NtAn1a* or *NtAn1b* gene (Bai et al., 2011). The early biosynthesis genes (EBGs), such as *CHS*, *CHI*, and *F3H*, and the late biosynthesis genes (LBGs) in the anthocyanin pathway, including *DFR* and *ANS*, were dramatically induced by the overexpression of *NtAn1* genes in tobacco (Bai et al., 2011). In this present paper, we found that the targeted mutagenesis of *NtAn1* genes generated white flower phenotype, which resulted from the defects in anthocyanin accumulation in the flower (Figure 5A). Previous study demonstrated that *TT8* gene regulated the biosynthesis of anthocyanin by manipulating the expression of the LBGs in anthocyanin pathway (Zhai et al., 2019). The expression level of the downstream genes at different flower development stages were analyzed by qRT-PCR. Our results revealed that the examined anthocyanin biosynthesis genes expressed at all three developmental stages with the expression level peaking at the late stage in WT plant flower (Figure 5B). The expression patterns of these genes were consistent well with those of *NtAn1* genes, which indicated the regulation relation between them (Bai et al., 2011). By contrast, the expression of all examined anthocyanin biosynthesis genes at different developing stages was significantly repressed in the *an1-1* mutant line (Figure 5B). Taken together, our results demonstrated again that *NtAn1* genes played an essential role in the biosynthesis of anthocyanin in tobacco flower.

### Targeted Mutagenesis of *NtAn1* Increased Seed Lipid and Protein Content

**Figure 6:**
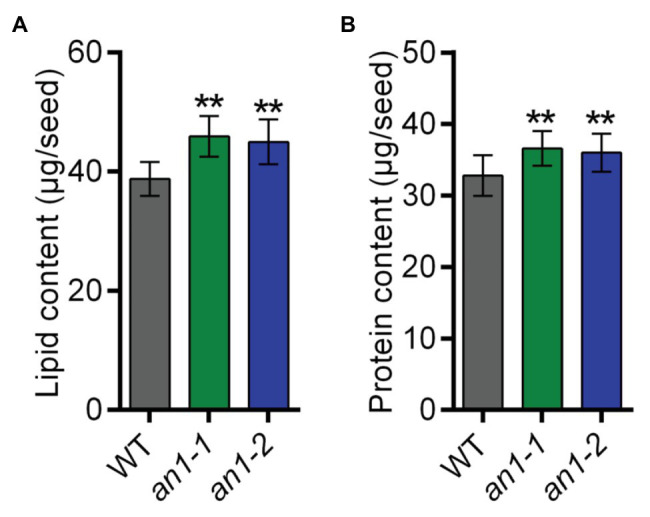
Lipid and protein content of the WT and *an1* mutant seeds. **(A)** Lipid content per seed. Twenty seeds were analyzed for each repeat and per seed lipid content was calculated by dividing the seed number. Data are mean ± SD (*n* = 3). **(B)** Protein content per seed. Ten seeds were analyzed for each repeat and per seed protein content was calculated by dividing the seed number. Data are mean ± SD (*n* = 3). Asterisks indicate significant difference compared to control samples (**p < 0.01).

Both natural and targeted mutation of *TT8* genes in *Arabidopsis* and *Brassica* species would result in a significant increase in seed lipid content. To characterize the effect of the *NtAn1* gene targeted mutation on tobacco seed lipid accumulation, the seed lipid content was analyzed by the GC-MS method. The results showed that WT tobacco seed lipid content was about 38.77 μg per seed, and the lipid content was approximately 45.91 μg per seed in *an1-1* and 44.97 μg per seed in *an1-2* mutant line, which increased significantly by 18.42 and 15.99% relative to the WT seeds, respectively (Figure 6A). These results indicated that the *TT8* gene and its homologs regulated seed lipid accumulation in a conserved way among different plant species. Thus, the *TT8* homologs from other oilseed plants could be used as a target to enhance the seed lipid content.

In most oilseed crops, the seed lipid content is negatively correlated with the protein content. Surprisingly, the *BnTT8* mutant seeds showed simultaneous increases in both lipid and protein contents (Zhai et al., 2019), which are different from the lower protein content in *Arabidopsis tt8* mutant seed (Chen et al., 2014). These results indicated that the seed protein accumulation was regulated by different mechanism in *Arabidopsis* and *B. napus*. To determine the effect of *NtAn1* genes on tobacco seed protein accumulation, the seed protein contents in WT and *an1* mutant lines were examined using the Pierce BCA Protein Assay Kit. The results showed that WT tobacco seed protein content was about 32.79 μg per seed, and the protein content was elevated to 36.56 μg per seed in *an1-1* and to 35.97 μg per seed in *an1-2* mutant line, increased significantly by 11.50 and 9.70% relative to the WT seeds, respectively (Figure 6B). These results were consistent with those in *B. napus* (Zhai et al., 2019). The seed meals of oilseeds are usually used as animal feed. Compared with the black-seeded rapeseed, the increased protein level makes the seed meal generated from yellow-seeded rapeseed more valuable for animal feed production (Bell, 1993; Jiang et al., 2015). The increased protein content of the *an1* mutant seeds could make the yellow-seeded tobacco seed meal more valuable for animal feed.

The property of biodiesel is partially determined by the fatty acid chain length, the position, and the number of the double bonds (Durrett et al., 2008). In *Arabidopsis* and *B. napus*, the mutation of *TT8* gene resulted in an alteration in the seed fatty acid profile, including increases in palmitic acid, linoleic acid, and linolenic acid and decreases in stearic acid and oleic acid compared with the WT seeds (Chen et al., 2014; Zhai et al., 2019). Fatty acid composition of tobacco seed lipid shows the main presence of palmitic acid, stearic acid, oleic acid, and linoleic acid (Giannelos et al., 2002). Possibly due the differences in the fatty acid composition, targeted mutation of *NtAn1* genes just resulted in a significant decrease in stearic acid, and the other four main fatty acid components were not changed significantly compared with the WT tobacco seed (Table 1).

**Table 1:** Fatty acid composition of the *an1* mutant lines.

	Fatty acid composition (mol%)				
	16:0	18:0	18:1^Δ9^	18:2^*Δ*9, 12^	18:3^Δ9, 12, 15^
WT	11.14 ± 0.37	4.36 ± 0.36	12.86 ± 0.35	71.14 ± 1.29	0.70 ± 0.02
*an1-1*	11.37 ± 0.28	2.77 ± 0.24^**^	13.02 ± 0.38	72.69 ± 1.43	0.98 ± 0.03
*an1-2*	11.96 ± 0.39	2.35 ± 0.21^**^	12.77 ± 0.29	73.08 ± 1.39	0.73 ± 0.01

*16:0, palmitic acid; 18:0, stearic acid; 18:1^Δ9^, oleic acid; 18:2^Δ9, 12^, linoleic acid; 18:2^Δ9, 12, 15^, linolenic acid. The data are the mean ± SD (n = 3)*. Asterisks indicate statistically significant differences, **p < 0.01.

### Expression of Genes Involved in Seed Development and Lipid Biosynthesis Were Altered by *NtAn1* Targeted Mutation

**Figure 7:**
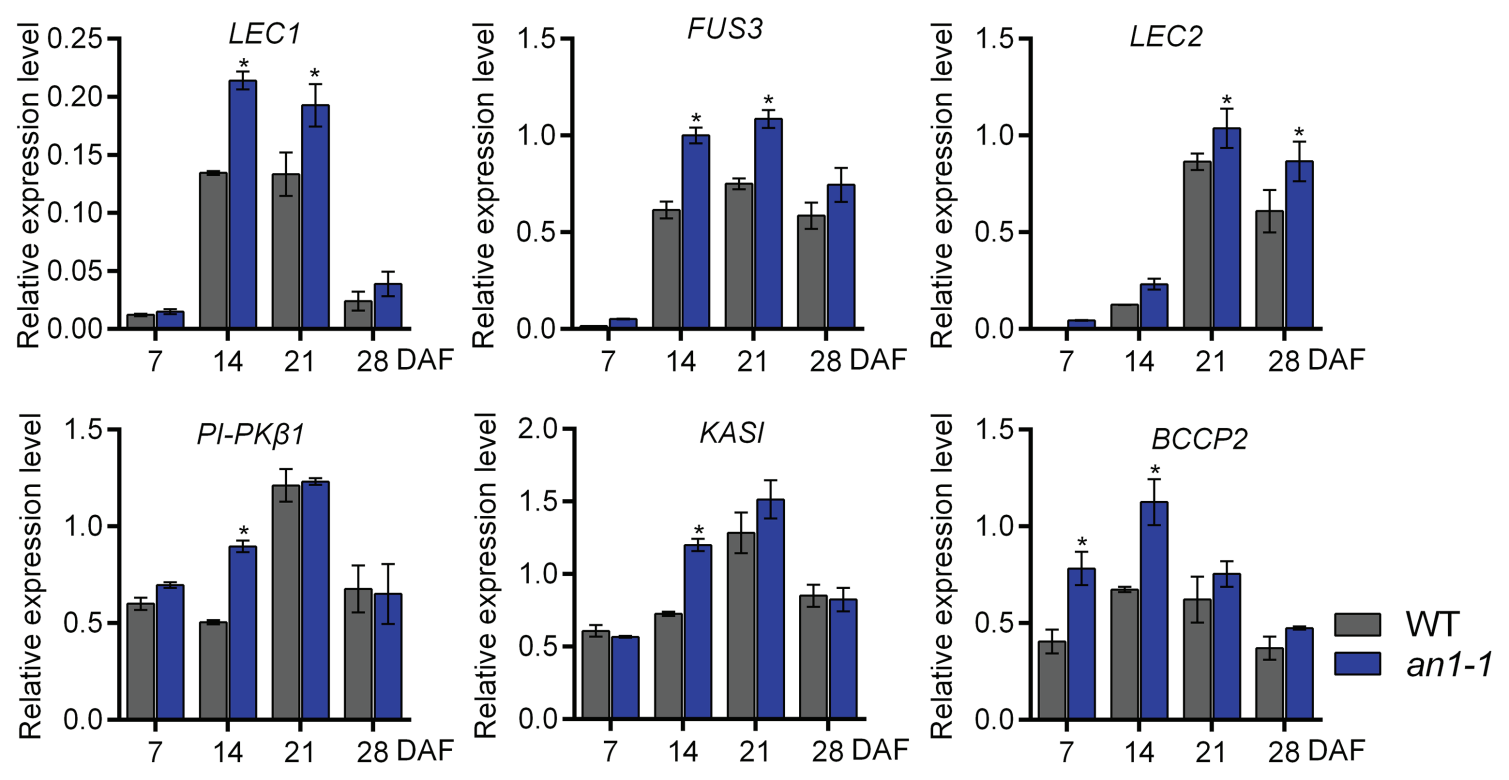
Expression analysis of genes involved in seed development and fatty acid biosynthesis. Asterisks indicate significant difference compared to control samples (*p < 0.05).

In *Arabidopsis*, TT8 protein could repress the lipid biosynthesis pathway by directly binding to the promoter region of the critical transcriptional factors, such as *LEAFY COTYLEDON1* (*LEC1*), *LEC2*, and *FUSCA3* (*FUS3*), that are important for seed development. In addition, TT8 gene could indirectly inhibit the expression of a number of important genes, including *KASII*, *MOD1*, *FAB2*, *FatA*, *FAE1*, *FAD2*, and *FAD3*, in the fatty acid biosynthesis pathway. Thus, the Arabidopsis *tt8* mutant showed increased seed lipid content (Chen et al., 2014). In *B. napus* L., the expression levels of several genes involved in fatty acid biosynthesis during seed development were increased in *BnTT8* mutant plants generated by the CRISPR-Cas9 system (Zhai et al., 2019). In this present paper, the targeted mutagenesis of *NtAn1* genes led to the increases of both lipid and protein content, so we suggested that *NtAn1* genes might regulate the seed development and storage component accumulation in a way similar to that in *B. napus*. To test this hypothesis, the expression of the transcription factors regulating seed development, including *LEC1*, *LEC2*, *FUS3*, and the enzymes that are important in the fatty acid biosynthesis pathway, such as *KASI*, *PI-PKβ1*, and *BCCP2*, were examined by qRT-PCR in the process of seed development. Our results showed that the expression of all examined genes was upregulated in the *an1-1* mutant at one or two developmental stages (Figure 7). For example, the *LEC1* and *FUS3* genes were significantly increased at 14 and 21 DAF seeds, while the *KASI* and *PI-PKβ1* genes were upregulated at 14 DAF stage (Figure 7). The elevated expression levels of genes involved in seed development and fatty acid biosynthesis could explain the enhanced lipid content in mutant lines.

### Seed Yield Related Traits Were Not Affected by Mutation of *NtAn1*

**Figure 8:**
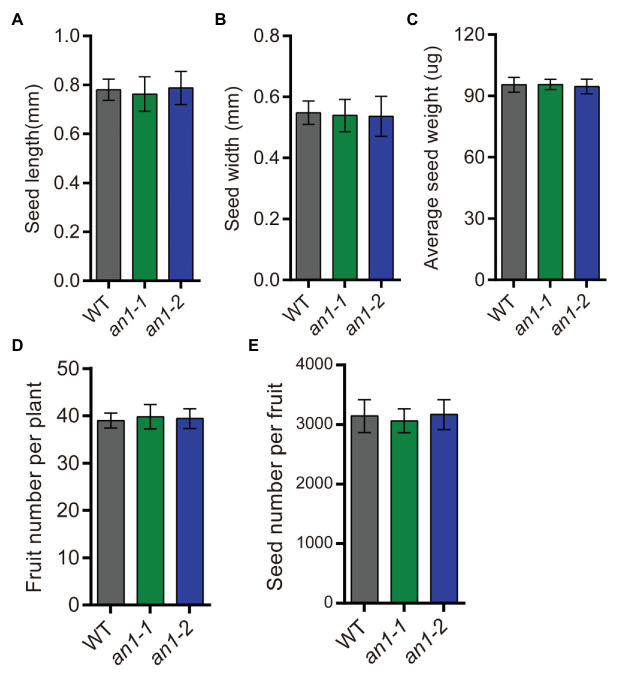
Seed yield related traits of *an1* mutant tobacco lines. **(A)** Average seed length. Data are mean ± SD (*n* = 30). **(B)** Average seed width. Data are mean ± SD (*n* = 30). **(C)** Average seed weight. Values are mean ± SD of five individual measurements of 50 seeds/replicate. The seeds used to measure seed size and seed weight were selected randomly after collecting all the mature seeds. **(D)** Fruit number per plant. Fruit number was calculated at the plant mature stage. Data are mean ± SD (*n* = 20). **(E)** Seed number per fruit. Data are mean ± SD (*n* = 10). Seed number per fruit was calculated from the basal five fruits at the mature stage.

Besides the seed lipid content, seed yield is another important factor affecting the lipid yield. The seed yield-related traits of the *an1-1* and *an1-2* mutant lines were also evaluated. The results showed that the yield-related traits, including seed size (Figures 8A,B), seed weight (Figure 8C), fruit number per plant (Figure 8D), and seed number per fruit (Figure 8E), were not affected by targeted mutation of *NtAn1* genes. Thus, the targeted mutation of *NtAn1* genes could generate a useful tobacco variety with a high seed lipid yield and improved nutritional quality.

## Discussion

### TT8 Homolog Genes Function in a Conserved Way to Regulate Seed Coat PAs and Seed Lipid Accumulation

**Figure 9:**
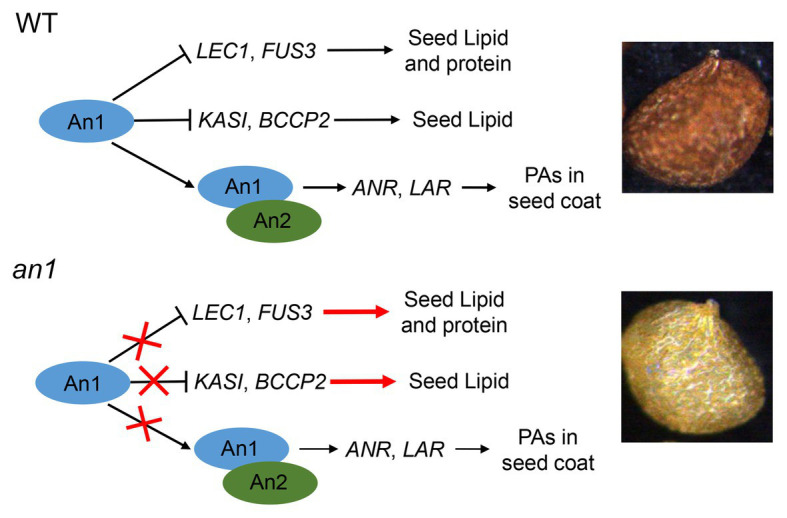
Model for An1-mediated regulation of PAs and lipid accumulation in tobacco seed. In WT tobacco seed, An1 protein could inhibit the expression of transcription factors (including *LEC1* and *FUS3*) and fatty acid biosynthesis genes (including *KASI* and *BCCP2*). Together with An2, a R2R3-MYB family protein, An1 could induce the expression of function genes (*LAR* and *ANR*) involved in PA biosynthesis in WT tobacco seed coat. In *an1* mutant seed, the inhibition on genes encoding transcription factor and fatty acid biosynthesis enzymes are released; thus, lipid and protein accumulation are enhanced in mutant lines. Compared with WT seed, the PA biosynthesis genes represent a lower expression level in mutant seeds and the PA accumulation is blocked, which lead to the yellow-seeded phenotype.

Compared with the oilseeds with a black seed coat, the oilseeds with a yellow-seeded phenotype showed a thinner seed coat, a reduced PAs content, and an increased content of lipid, thus a yellow-seeded phenotype is a preferred trait for numerous oilseed plant genetic breeding (Rahman and Mcvetty, 2011). Due to the accumulation of PAs in the seed coat, tobacco seed showed a black color. Previous studies had demonstrated that the TT8 protein played a key regulation role in PA biosynthesis in seed coat (Xu et al., 2014). Two *TT8* homologs, *NtAn1a* and *NtAn1b*, had been identified in tobacco genome (Bai et al., 2011). However, the functions of seed color formation and seed lipid accumulation of these two genes had not yet been characterized. In this paper, we found that *NtAn1* genes were highly expressed in developing seeds (Figure 1), which indicated that they might play a role in seed development. A yellow-seeded phenotype was generated by CRISPR-Cas9-mediated targeted mutagenesis of tobacco *NtAn1* genes (Figure 3A). DMACA staining of seed coat and extraction analysis further confirmed that the mutation of *NtAn1* genes blocked the specific PA deposition in the seed coat (Figures 3C,D). Most importantly, seed lipid and protein contents were both dramatically increased by targeted mutagenesis of *NtAn1* genes (Figure 6). In *Arabidopsis*, TT8 induces the expression of LBGs of PAs through directly binding to the regulatory region (Xu et al., 2014). Natural mutation of *BrTT8* by a large insertion resulted in yellow seed coat in *Brassica rapa*, and the LBGs were significantly downregulated by *BrTT8* mutation (Li et al., 2012). In this paper, the LBGs, including *ANR* and *LAR*, were significantly decreased in both *an1-1* and *an1-2* mutant lines (Figure 3D). In *Arabidopsis*, TT8 protein could also directly bind to the promoter region of transcription factors that are important for seed development and lipid biosynthesis and repress the expression of them (Chen et al., 2014). In this paper, the expression of the downstream transcription factors and fatty acid biosynthesis genes were significantly upregulated during seed development in the *an1-1* mutant line (Figure 7). Most recently, similar phenomena were observed in CRISPR-Cas9-mediated *TT8* gene mutation in *B. napus* (Zhai et al., 2019). Previous reports and results in this present paper indicated that *TT8* homolog gene functions in a conserved way in seed coat PAs and seed lipid biosynthesis regulation. Based on the results in this present paper, a model for NtAn1 protein-mediated regulation of PAs and lipid accumulation was constructed (Figure 9). In WT tobacco seed, the NtAn1 protein could inhibit the expression of transcription factor (including *LEC1* and *FUS3*) and genes encoding fatty acid biosynthesis enzyme (including *KASI* and *BCCP2*). Due to the inhibition effect of NtAn1, WT seeds showed a lipid content of about 38 μg per seed (Figure 6A), and a protein content of about 32 μg per seed (Figure 6B). By contrast, in *an1* mutant seed, the inhibition on transcription factors and fatty acid synthesis genes are released, thus lipid and protein accumulation are elevated to about 45 and 36 μg per seed, respectively. Together with An2, a R2R3-MYB family protein, An1 could induce the expression of function genes (*LAR* and *ANR*) involved in PAs biosynthesis in WT tobacco seed coat. The accumulation of PAs in seed coat generates the black-seeded phenotype in WT seed. However, the PAs biosynthesis genes represent a lower expression level in mutant seeds and the PA accumulation is blocked, which lead to the yellow-seeded phenotype (Figure 9). PAs are widely observed in seed coat in a number of oilseed plants, such as *Camelina sativa*, *Camellia oleifera*, and Tree peony. We proposed that *TT8* homolog genes could be used as an ideal target for enhancing seed lipid accumulation in these plants.

### CRISPR-Cas9 System Could Be Used for *de novo* Tobacco Domestication for Biodiesel Feedstock Production

In recent years, the CRISPR-Cas9-based sequence-specific nucleases (SSNs) had been demonstrated to be the most simple and efficient tool for targeted gene editing. The CRISPR-Cas9 system has been successfully utilized in tobacco to generate the required mutagenesis for agronomic traits improvement and gene function characterization (Jiang et al., 2013; Gao et al., 2018; Schachtsiek and Stehle, 2019). Previous reports had demonstrated that homozygous mutant could be obtained in the first generation in diverse plants, including tomato, rice, grape, and poplar (Zhang et al., 2014; Fan et al., 2015; Pan et al., 2016; Wang et al., 2018). In this present paper, two homozygous mutant lines were generated in the first generation (Figure 2); our results demonstrated again that the CRISPR-Cas9 system is a highly efficient method for targeted gene mutation. The mutant lines showed an expected increase in seed lipid content (Figure 6A). In addition, the seed yield was not compromised (Figure 8).

Due to the ever-increasing global need for energy and environmental concerns about the effects of increasing carbon dioxide levels, the demand for biofuels has been dramatically increased in the past decades (Durrett et al., 2008). To meet the huge demand for biofuel feedstocks, *de novo* domestications of plants for biofuel production have attracted a lot of attention worldwide (Montes and Melchinger, 2016; Sainger et al., 2017). Tobacco seed oil is a promising feedstock for biodiesel production; however, current tobacco varieties are not bred for seed lipid production. With the development of CRISPR-Cas9 system, it has been proposed that CRISPR-Cas9-mediated genome editing could be used as a new tool by breeders to accelerate the domestication of semi-domesticated or even wild plants (Osterberg et al., 2017; Fernie and Yan, 2019; Khan et al., 2019). Low linoleic acid and high oleic acid content is a preferred character for biodiesel feedstock production, so we created a high-oleic acid tobacco variety using CRISPR-Cas9-mediated *NtFAD2-2* gene editing technology in our previous work (Tian et al., 2020). In the past few years, CRISPR-Cas9-mediated domestication had been carried out in a number of wild plant species, such as wild tomato and groundcherry (Zsogon et al., 2017, 2018; Lemmon et al., 2018; Li et al., 2018).

Tobacco belongs to the Solanaceae family, which contains several well-characterized model crops, including tomato, potato, and pepper. Numerous regulators important for yield related traits, including fruit size, inflorescence, and shoot architecture, had been identified and characterized in these model crop species, especially tomato (Rothan et al., 2019). These genes include *SP* (*SELF-PRUNING*; Pnueli et al., 1998), *fw2.2* (*FRUIT WEIGHT 2.2*; Frary et al., 2000), *FASCIATED* (Xu et al., 2015a), and *MULTIFLORA* (Lippman et al., 2008). Previous studies had demonstrated that the genetic regulation networks of agronomic traits were conserved in different plant taxa, which suggested that editing the homolog genes across species may generate similar phenotypes (Hussain et al., 2020). We suggested that the domestication knowledge from model crops could be translated into tobacco for the generation of high seed yield and high lipid content varieties. In this paper, seed lipid content was significantly increased by targeting a single gene and the multiplex CRISPR-Cas9 system could be applied in the future to simultaneously target several genes for multiple traits enhancement.

## Conclusion

In this study, we showed that *NtAn1a* and *NtAn1b* genes were highly expressed in developing tobacco seed. Targeted mutation of *NtAn1* genes were generated using the CRISPR-Cas9-mediated genome editing technology. Due to the defects in PAs biosynthesis, the mutant seeds showed the yellow-seeded phenotype. We showed that targeted mutagenesis of *NtAn1* genes enhanced the seed lipid accumulation by about 18% than WT control seeds. The high knockout efficiency and significantly elevated lipid content in mutant seeds indicated that the CRISPR-Cas9 system could be applied to generate new tobacco varieties for biodiesel production in a faster way than traditional breeding method.

## Data Availability Statement

The original contributions presented in the study are included in the article/Supplementary Material, further inquiries can be directed to the corresponding author.

## Author Contributions

YT and YX conceived and designed the study and wrote the manuscript. YT and XL performed the experiments. CF and YZ contributed in manuscript revision. TL, HQ, XL, and KC contributed to data acquisition. All authors reviewed, discussed, and interpreted the results. FC reviewed the manuscript. All authors contributed to the article and approved the submitted version.

### Conflict of Interest

The authors declare that the research was conducted in the absence of any commercial or financial relationships that could be construed as a potential conflict of interest.

## Footnotes

^1^http://crispr.hzau.edu.cn/CRISPR2/
