# Supplementary material for: Enhancement of Tobacco (Nicotiana tabacum L.) Seed Lipid Content for Biodiesel Production by CRISPR-Cas9-Mediated Knockout of NtAn1
Source: Front Plant Sci. 2021 Jan 21;11:599474. doi: 10.3389/fpls.2020.599474 (PMC7859101; doi:10.3389/fpls.2020.599474)
Supplement: Supplementary file 1 [file Table_1.doc]

**Supplementary Figures and Tables.**


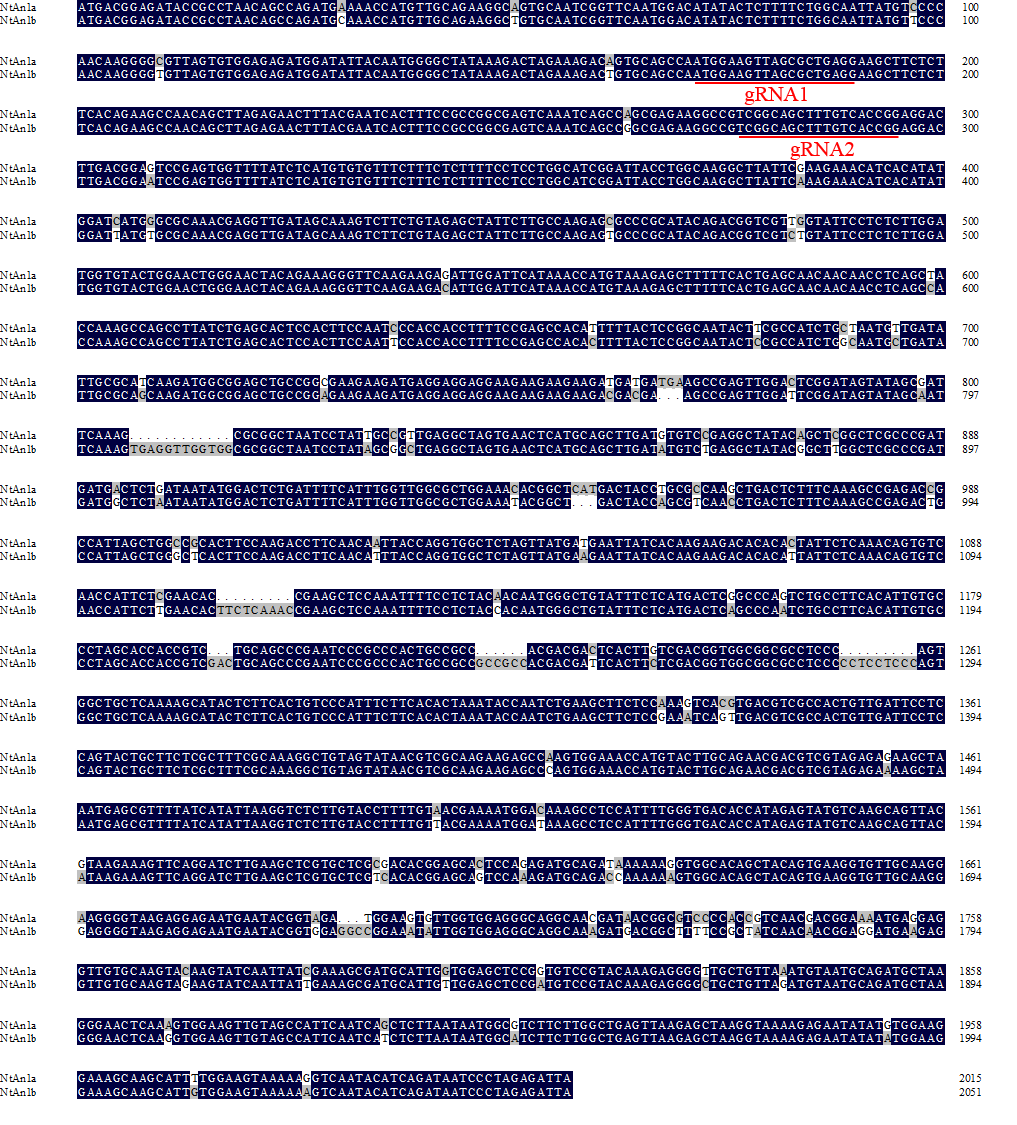


**Figure S1.** Protein coding sequence alignment of *NtAn1a* and *NtAn1b*. The two gRNAs targeting sites are underlined.


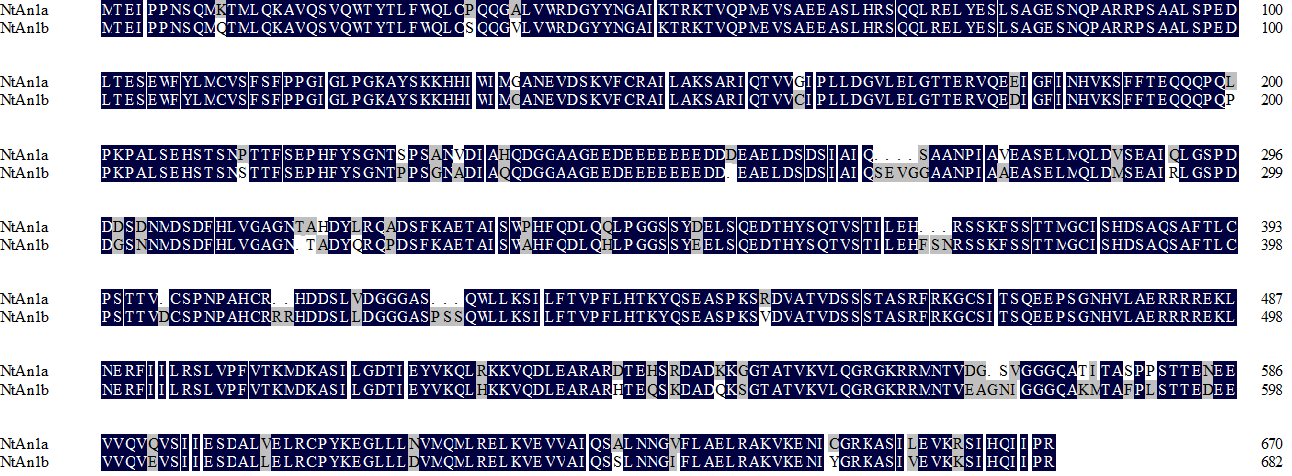


**Figure S2.** Protein sequence alignment of NtAn1a and NtAn1b.

**Table. S1** Primers used for qRT-PCR analysis in this paper.

| Genes name | Forward primer sequences (5′–3′) | Reverse primer sequences (5′–3′) |
| --- | --- | --- |
| *NtAn1a* | GCGATTCAAAGCGCGGCTAATC | CAGCTTGGCGCAGGTAGTCATG |
| *NtAn1b* | AGTGAGGTTGGTGGCGCGGC | CAGGTTGACGCTGGTAGTCAG |
| *ANR* | CGGACGATACATTTGTTCTGC | TGATCAACTTGGCCTTGGAG |
| *LAR* | AGGAGATGCTAAGGAAACATGAG | GGATCTGACCTGTCTACTTCATG |
| *CHS* | GCCGCAATCATTATAGGTTCTG | TGTTAGCCCAACTTCACGTAG |
| *F3H* | TTGGATTACTGTTCAGCCCG | GGTACACTATAGCTTCTGGTGC |
| *ANS* | CCGAAAACCCCTACAGACTAC | TCTCAGCCCAATAGAAAGCAC |
| *DFR* | TCAAGGCTGTCAAGGAGTATTC | TGCTTAACATTCCCCTGACTG |
| *LEC1* | TGGTGAGCGTGGATCTTTG | TGCATATAACCTTTCCCCGTC |
| *FUS3* | CCGACAGGGTTCAGATTTCTC | CTCAAGAGTTGGGAGATATCGC |
| *LEC2* | CTTGAGAACACAGGCGATTTTG | TCCACTCTGCCTCACTTTTAC |
| *KAS1* | GCAACCAAGTCTATGATAGGGC | TCCGACATTCACTTCATGCTG |
| *PI-PKβ1* | TGAGCCATTATCGTCCTTGTG | TTCACCATCCCTTGCTTCTG |
| *BCCP2* | CCTCCACTCAAATGTCCCATG | CCTCCACTACAGTTCCAGATTG |

**Table. S2** Primers used for targeted gene editing in this paper.

| Primers name | Sequences (5′–3′) | Used for |
| --- | --- | --- |
| An1-DT1-BsF | ATATATGGTCTCGATTGCCTCAGCGCTAACTTCCATGTT | CRISPR-Cas9 vector construction |
| An1-DT1-F0 | TGCCTCAGCGCTAACTTCCATGTTTTAGAGCTAGAAATAGC |
| An1-DT2-R0 | AACTCGGCAGCTTTGTCACCGGCAATCTCTTAGTCGACTCTAC |
| An1-DT2-BsR | ATTATTGGTCTCGAAACTCGGCAGCTTTGTCACCGGCAA |
| pANR-F | TCACGCGTTCGCGAATCGATCCGCGGGACATGTAGACCGGGGAAACTAAC | Yeast one-hybrid system vector construction |
| pANR-R | TAATGCCAGGAATTTCTAGACCGCGGGGATTCTTAATGTTTTTAAAGTCAG |
| An1-F | ATGGCCATGGAGGCCAGTGAATTCATGACGGAGATACCGCCTAACAGC |
| An1-R | CTGCAGCTCGAGCTCGATGGATCCTTAATCTCTAGGGATTATCTGATG |
| An2-F | AAAATATACCCCAGCCTCGAGATGAATATTTGTACTAATAAGTC |
| An2-R | AAGAAGTCCAAAGCTGGATCCTCAACTGAGAAGTGGCATTTCCTC |
| Box-An2-F | CGACTCACTATAGGGCGAATTCCAAGCTTCCTGAAACGGAGAAAC |
| Box-An2-R | CGATTCGCGAACGCGTGAGCTCGCCGGTAGAGGTGTGGTCAATAAG |
| ge-NtAn1-F | GCAATCGGTTCAATGGACATATAC | Target flanking sequence amplification |
| ge-NtAn1-R | GAACTTACCCGATGCCAGGAG |
| Cas9-F | GACCACGACGGGGATTACAAGG | Cas9 gene fragment amplification |
| Cas9-R | GGTGTTCACCCTGAGAATATCAG |
